# Overview on and Contextual Determinants of Medical Residencies in North Brazil

**DOI:** 10.3390/healthcare11081083

**Published:** 2023-04-11

**Authors:** Rafael Alves Guimarães, Ana Luísa Guedes de França e Silva, Alessandra Vitorino Naghettini, Heliny Carneiro Cunha Neves, Fernanda Paula Arantes, Cândido Vieira Borges Junior, Antônio Isidro da Silva Filho, Alessandra Rodrigues Moreira de Castro

**Affiliations:** 1 Nursing School, Federal University of Goiás, Goiânia 74690-900, Brazil; 2 Tropical Pathology and Public Health Institute, Federal University of Goiás, Goiânia 74690-900, Brazil; 3 Center for Innovation in Education and Health Work Management, Federal University of Goiás, Goiânia 74690-900, Brazil; 4 Medical School, Federal University of Goiás, Goiânia 74690-900, Brazil; 5 Business, Accounting, and Economic Sciences Schools, Federal University of Goiás, Goiânia 74690-900, Brazil; 6 Business, Accounting, Economics, and Public Management Schools, University of Brasília, Brasília 70910-900, Brazil; 7 Ministry of Health, Brasília 70058-900, Brazil

**Keywords:** medical residency, residencies, medical specialties, structural determinants of health

## Abstract

The aim of this study was to analyze the scenario of medical residency programs (MRPs) in the north region of Brazil as well as the contextual determinants (socioeconomic, structural, and epidemiological) influencing the number of MRPs in this region. An ecological study was conducted using MRPs data from 2022. This study used multiple data sources. MRP indicators were described based on the Brazilian state and specialty. The dependent variable was the number of MRPs. The independent variables included sociodemographic, structural, and epidemiological factors. Poisson regression was performed to analyze the association between contextual variables and the number of MRPs. The results showed that only 3.6% of the municipalities had authorized MRPs. The idleness rate in the region was 46.0%, with family and community medicine as the specialties with the greatest idleness. The total density of authorized vacancies in the MRPs was 14.0 vacancies per 100,000 inhabitants. The models showed that with each increase of one unit of the vulnerability index (Socioeconomic Index in the Geographic Context for Health Studies—GeoSES), the number of MRPs increased, ranging from 8122 (*p* value < 0.001) to 11,138 (*p* value < 0.001). With each increase in undergraduate degrees in medicine, the number of MRPs increased by 0.945 (*p* value < 0.001). With each increase of 1 physician per 1000 population, the number of MRPs increased from 0.537 (*p* value < 0.001) to 0.845 (*p* value < 0.001). With each increase of one unit in general hospitals, specialized hospitals, teaching hospitals, and primary healthcare units, the number of MRPs increased by 0.176 (*p* value < 0.001), 0.168 (*p* value < 0.001), 0.022 (*p* value < 0.001) and 0.032 (*p* value < 0.001), respectively. Finally, with each increase of one death per 100,000 inhabitants, the overall mortality rate increased, ranging from 0.006 (*p* value < 0.001) to 0.022 (*p* value < 0.001). The study showed a low supply of MRPs in the northern region, a high rate of idleness, and important socioeconomic, structural, and epidemiological determinants of the number of MRPs.

## 1. Introduction

Medical residency programs (MRPs) are defined as postgraduate teaching modalities used to train and develop new specialists in multiple medical fields. These programs, which have been implemented in Brazil since 1944, are featured as training models aimed at providing professional specialization, with emphasis on in-service training. It is considered the best strategy for training new specialists and qualifying physicians [1,2].

Medical residency was formalized in Brazil in 1977 and regulated by Law No. 6932 on 7 July 1981 [3]. In 1977, the National Medical Residency Commission (in Portuguese, Comissão Nacional de Residência Médica—CNRM), which is linked to the Ministry of Education (in Portuguese, Ministério da Educação, MEC), accounted for MRP accreditation and re-accreditation processes as well as regulating, supervising, and assessing program institutions. In addition to MEC, it comprises members of the Ministry of Health (in Portuguese, Ministério da Saúde—MS), the National Council of Health Secretariats (in Portuguese, Conselho Nacional de Secretários de Saúde—CONASS), the National Council of Municipal Health Secretariats (in Portuguese, Conselho Nacional de Secretarias Municipais de Saúde—CONASEMS), the Federal Council of Medicine (in Portuguese, Conselho Federal de Medicina—CFM), the Brazilian Association of Medical Education (in Portuguese, Associação Brasileira de Educação Médica—ABEM), the Brazilian Medical Association (in Portuguese, Associação Médica Brasileira –AMB), the National Association of Resident Physicians (in Portuguese, Associação Nacional de Médicos Residentes—ANMR), the National Federation of Physicians (in Portuguese, Federação Nacional dos Médicos—FENAM), and the Brazilian Federation of Medical Academies (in Portuguese, Federação Brasileira de Academias de Medicina—FBAM) [4]. Furthermore, the State Medical Residency Commission (in Portuguese, Comissão Estadual de Residência Médica—CEREM) is subordinate to CNRM and has decision-making power over residency matters in Brazil. In addition, it guides the Medical Residency Commission (in Portuguese, Comissão de Residência Médica—COREME) of institutions belonging to programs that account for planning, coordinating, supervising, and assessing institutions’ MRPs [5].

After the implementation of the Brazilian Unified Health System (in Portuguese, Sistema Único de Saúde—SUS) in 1988, the MS and the National Health Council (in Portuguese, Conselho Nacional de Saúde—CNS) were responsible for organizing training for human resources in this field [1]. In 1989, CNRM lost its exclusivity in the process of providing specialist titles, as CFM and AMB started to acknowledge physicians who received this title through examinations held by several medical specialty societies as specialists [1]. In 2002, MEC, CFM, and AMB began to share attributions in the acknowledgement of specialties: CNRM authorizes MRPs, AMB supervises specialist title granting, and CFM registers these titles [6].

In recent years, the number of vacancies has significantly increased, driven by different programs and strategic policies. In 2009, MEC and MS implanted the National Support Program for Specialist Physicians’ Training in Strategic Fields (in Portuguese, Programa Nacional de Apoio à Formação de Médicos Especialistas em Áreas Estratégicas—Pró-Residência). This program was operationalized by increasing the offer of scholarships in MRPs, with emphasis on regions and specialties experiencing the largest gaps in training and care. Its features include scholarship-granting policy for residents; institutional support to open and expand the number of vacancies in priority SUS regions and fields; and training managers, preceptors, and tutors to work in MRPs [1,7]. In 2013, Law No. 12,871 created the More Doctors Program (in Portuguese, Programa Mais Médicos—PMM), now renamed the Doctors in Brazil Program (in Portuguese, Programa Médicos pelo Brasil—PMB), to train medical human resources for SUS. It presented actions focused on reorganizing the offer of vacancies for medical courses and MRPs, with an emphasis on health regions presenting a lower number of vacancies and physicians per inhabitant ratio, in addition to other criteria [8].

In 2019, CFM approved Ordinance number 1/2018, which updated the list of medical specialties and practice fields. The ordinance approved a list of 55 specialties and 59 areas of expertise [9]. In 2019, a study showed that 56,255 vacancies in MRPs were authorized. An idleness rate of 29.3% was verified. Only 5% of the cities in Brazil offer vacancies in MRPs. The MS and State Health Secretariat are the largest funders of MRPs in Brazil. Together, they invested R$ 129 million in grants [1].

Despite advancements in policies focused on expanding the number of vacancies in MRPs, this process did not occur homogeneously in the national territory. The distribution of vacancies is followed by strong socioeconomic and demographic differences among the Brazilian regions. Based on 2019 data, the least developed regions (North and Northeast) received little incentive to open vacancies, leading to inequality in the offer of specialists in these regions. The North region accounts for the smallest number of MRPs, as well as the highest idleness rates and the lowest funding for residency grants. In 2019, only 16 (4.0%) of the 450 municipalities in the region had MRPs. However, the Southeast and South regions account for the largest number of offered vacancies and for the largest number of preceptors and hospital beds, as well as the largest number of hospital facilities and technologies to facilitate MRPs expansion and implementation. Thus, it is evident that the least developed regions have a harder time retaining and attracting medical professionals to MRPs [1].

The implementation of the MRP is a complex process. Multiple CNRM regulations have established the minimum requirements for MRP accreditation in institutions. Examples include knowing the current legislation, having ethical-legal support, having professionals such as qualified preceptors and supervisors, having basic support services with sufficient human resources comprising adequate personnel, and having educational and scientific programming in regular operation [10]. Thus, both the presence and the number of MRPs in a given region are strongly influenced by indicators such as institutional infrastructure conditions and qualification of both the pedagogical project and the teaching staff, that is, of preceptors and supervisors [11]. In addition to proper infrastructure, the process of opening and selecting MRPs’ medical specialties should be compatible with the socioeconomic and epidemiological profile of the implementation region [1].

Contextual factors such as the number of general and specialized hospitals as well as of primary care health units, indicators that can be used as basic infrastructure proxy and practice fields, the presence of medical undergraduate courses, a vulnerability index, and local mortality and hospitalization rates can all influence both the presence and the number of MRPs in the most diverse cities. Nevertheless, no studies focusing on investigating the association between contextual socioeconomic factors, basic infrastructure, and epidemiological factors and the presence/number of MRPs in Brazil were found in the literature. Existing studies focusing on tracing the MRPs scenario are limited to describing key indicators, such as number of vacancies, resident density, and idleness rate [1,12], without considering other contextual determinants (socioeconomic, structural, and epidemiological).

It is essential to conduct studies focused on analyzing the scenario and contextual determinants of MRPs in the North region to help improve public policies aimed at expanding and improving the programs implemented in it, in compliance with the National Plan for Strengthening Residencies in the Health Field (in Portuguese, Plano Nacional de Fortalecimento das Residências em Saúde—PNFRS) [13]. This plan was implemented in 2021 by the MS through its Labor Management and Health Education Secretariat (in Portuguese, Secretaria de Gestão do Trabalho e da Educação na Saúde–SGTES). It comprises a set of strategic actions structured in three axes to value the qualifications of residents, assistance faculty, residency program managers, and institutional support for MRPs operating in the country. One of its aims is to monitor the number, quality, and field of MRPs implemented in Brazil [13].

It should be noted that the first cycle of the PNFRS prioritized the northern region by providing institutional support for the creation, reactivation, and restructuring of MRPs. Thus, these determinants play an important role in outlining strategies focused on valuing and retaining residents in these places, as well as in investigating factors capable of influencing the implementation of strategic MRPs. Therefore, the present study aimed to analyze the scenario of MRPs in the northern region, as well as the contextual determinants (socioeconomic, structural, and epidemiological) influencing the number of MRPs in this region.

## 2. Methods

### 2.1. Design

An ecological study focused on analyzing the MRPs distribution scenario and its association with contextual determinants (socioeconomic, structural, and epidemiological) in northern Brazil.

### 2.2. Setting

**Figure 1:**
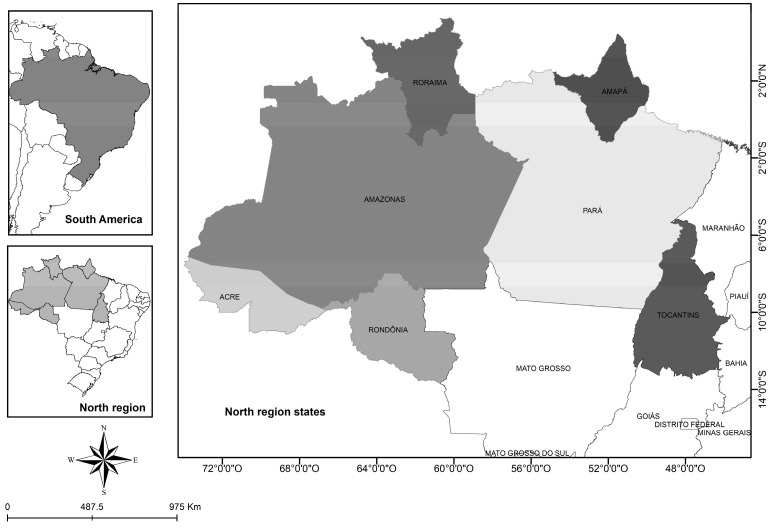
Study scenario—states in the North region of Brazil.

The current study analyzed data on the MRP in the northern region of the country, with a territorial area of 3,853,676,948 km^2^ and corresponding to 45.3% of the national territory. Its Human Development Index (HDI) is 0.730, and it comprises seven states: Acre, Amapá, Amazonas, Pará, Rondônia, Roraima, and Tocantins. The northern region comprises 450 cities. The region’s estimated population comprises 18,672,591 inhabitants, and its Gross Domestic Product (GDP) per capita is R$21,323.93 [14]. Figure 1 shows a location map of the states in northern Brazil.

The timeframe from 1 January to 17 October 2022, was used for MRPs analysis, as well as contextual indicators from 2010 to 2020 (last periods available for analysis).

### 2.3. Data Source

All data used in the current study were open access and were available online. The following data sources were used in the present research: (i) CNRM database of the MEC was used to extract MRPs data made available through the Integrated Ombudsman and Access-to-Information Platform; (ii) database of the intercensal estimates for the population carried out by the Brazilian Institute of Geography and Statistics (in Portuguese, Instituto Brasileiro de Geografia e Estatística—IBGE) accessed by United Nations Program for Human Development in Brazil (in Portuguese, Programa das Nações Unidas para o Desenvolvimento Humano do Brasil—PNUD) [15]; (iii) 2010 IBGE demographic census [15]; (iv) Socioeconomic Index database in the Geographical Context for Health Studies (GeoSES) [16]; (v) database of the National Register of Health Establishments (in Portuguese, Cadastro Nacional dos Estabelecimentos de Saúde—CNES) accessed by Departamento de Informática do SUS (in Portuguese, Departamento de Informática do SUS—DATASUS) [17]; (vi) database of the Higher Education Census of Anísio Teixeira National Institute of Educational Studies and Research (in Portuguese, Instituto Nacional de Estudos e Pesquisas Educacionais Anísio Teixeira—Inep) [18]; (vii) database of the Mortality Information System (in Portuguese, Sistema de Informação sobre Mortalidade—SIM) accessed by DATASUS [19]; and (viii) database of the Hospitalization System of the Unified Health System (in Portuguese, Sistema de Informações Hospitalares do Sistema Único de Saúde—SIH-SUS) accessed by DATASUS [20].

### 2.4. Indicators

The following MRPs data were extracted: (i) number of MRPs authorized in Brazil; (ii) number of vacancies authirized in MRPs; (iii) number of vacancies authorized, based on specialty or area of expertise, considering the specialties and areas of expertise officially acknowledged by the Mixed Specialties Commission (in Portuguese, Comissão Mista de Especialidades—CME) formed by CNRM, CFM and AMB; (iv) number of vacancies taken in MRPs; and (v) number of idle vacancies. Variables were calculated based on using CNRM/MEC data [21,22]. Only MRPs approved through CNRM/MEC authorization acts were included in the current study.

The following indicators were calculated:

(i) Density of authorized vacancies per 100,000 inhabitants based on the following formula:
  $$Density=\frac{Number\;of\;vacancies\;in\;MRPs\;authorized}{Total\;resident\;population}\times 100,000$$
(ii) Density of authorized vacancies of R1 (for the first year of residence) per 100,000 inhabitants based on the following formula:
  $$Density=\frac{Number\;of\;authorized\;vacancies\;of\;R1}{Total\;resident\;population}\times 100,000$$
(iii) Density of residents per 100,000 inhabitants based on the following formula:
  $$Density=\frac{Number\;of\;vacancies\;taken\;in\;MRPs}{Total\;resident\;population}\times 100,000$$
(iv) The idleness rate based on the following formula:
  $$Rate=\frac{Number\;of\;vacancies\;taken\;in\;MRPs}{Number\;of\;vacancies\;in\;MRPs\;authorized}\times 100$$
(v) Number of authorized MRPs, defined as the absolute number of programs authorized to operate in Brazil.

Table 1 shows the contextual indicators (socioeconomic, structural, and epidemiological) that were extracted for each city to enable the analysis of the association between these indicators and the number of MRPs.

The number of MRPs authorized in the city was used as the dependent variable. Contextual (socioeconomic, structural, and epidemiological) indicators were used as the independent variables.

### 2.5. Statistical Analysis

Data were analyzed using R software, version 3.3.3 for Windows [23]. Initially, the Kolmogorov–Smirnov test with Lilliefors correction was performed to verify the normality of the dependent and independent variables. For these variables, the mean, standard deviation, median, 25th percentile (P25), 75th percentile (P75), and minimum and maximum values were described. In addition, qualitative variables of the MRPs were described as absolute and relative frequencies.

Bivariate and multiple Poisson regressions were performed for inferential analysis. The number of municipalities in the northern region (*n* = 450) was used as the unit of analysis. Bivariate regression was performed to investigate the association between each contextual independent variable and dependent variable (number of MRPs). Variables showing *p* < 0.20 were included in the multiple Poisson regression model with robust variance. Multicollinearity analysis between variables was performed using Pearson’s correlation coefficients in pairs [24]; pairs showing coefficient ≥0.60 were considered collinear variables. Separate individual models were adjusted for collinear variables in the case of multicollinearity. *p* < 0.05 was considered statistically significant in all analyses.

### 2.6. Ethical Aspects

This study was approved by the Research Ethics Committee of the Federal University of Goiás (protocol 4, 675, 978/2021).

## 3. Results

In total, 295 MRPs were included in this study. Of the 450 cities in the northern region, only 16 (3.6%) had authorized MRPs. The distribution of the number of MRPs among the 16 cities and their respective states was as follows: Belém, Pará (*n* = 80, 27.1%), Manaus, Amazonas (*n* = 74, 25.1%), Palmas, Tocantins (*n* = 31, 10.5%), Porto Velho, Rondônia (*n*= 30, 10.2%), Rio Branco, Acre (*n* = 18, 6.1%), Santarém, Pará (*n* = 15, 5.1%), Boa Vista, Roraima (*n* = 11, 3.7%), Araguaína, Tocantins (*n* = 10, 3.4%), Macapá, Amapá (*n* = 10; 3.4%), Ananindeua, Pará (*n* = 3, 1.0%), Cacoal, Rondônia, (*n* = 3, 1.0%), Vilhena, Rondônia (*n* = 3, 1.0%), Gurupi, Tocantins (*n* = 3, 1.0%), Bragança, Rondônia (*n* = 2, 0.7%), Coari, Amazonas (*n* = 1, 0.3%), and Porto Nacional, Tocantins (*n* = 1, 0.3%). Cities of state capitals accounted for 86.1% (*n* = 254) of the MRPs, while those from cities outside the capital accounted for only 13.1% (*n* = 41).

Fifty-five institutions were accredited and authorized to offer vacancies in the MRP in the North Region, mostly in the states of Pará (*n* = 100, 33.9%) and Amazonas (*n* = 75, 25.4%). The MRPs considered 2261 authorized vacancies, of which 43.5% (*n* = 983) were R1 vacancies, that is, the first year of residency. Most vacancies were offered in the state of Pará (*n* = 911, 40.3%), followed by Amazonas (*n* = 729, 32.2%) (Table 2).

The idleness rate in the region was 46.0%; that is, almost half of the vacancies were not occupied, with higher percentages in Amapá (51.5%) and Roraima (54.2%). The lowest percentage was observed in Pará (26.2%) (Table 2).

The total density of authorized vacancies in MRPs in the northern region was 14.0 vacancies per 100,000 inhabitants in 2022. The highest density was found in Acre (20.1 vacancies/100,000 inhabitants) and the lowest in Pará (10.1 vacancies/100,000 inhabitants). Considering only the R1 vacancies (first year), the highest density remains in Acre (7.5 vacancies/100,000 inhabitants) and the lowest in Amapá and Pará (3.8 vacancies/100,000 inhabitants) (Table 2).

In 2022, there were 1622 residents enrolled in MRPs in the northern region, with the majority in the state of Pará (*n* = 672, 41.4%). The density of registered residents was 8.5 residents/100,000 inhabitants, with the highest density in Acre (11.9 residents/100,000 inhabitants) (Table 2).

Family and community medicine (*n* = 404, 17.97%), medical clinics (*n* = 292, 12.91%), pediatrics (*n* =275, 12.16%), and gynecology and obstetrics (*n* = 276, 12.21%) were the specialties/areas of expertise with the largest number of vacancies, whereas medical clinics had the highest number of residents (*n* = 237, 14.61%). Family and community medicine (2.13 residents/100,000 inhabitants), medical clinics (1.54 residents/100,000 inhabitants), gynecology and obstetrics (1.45 residents/100,000 inhabitants), pediatrics (1.45 residents/100,000 inhabitants), and general surgery (1.01 residents/100,000 inhabitants) were the specialties/areas of expertise with the highest density of residents. Idleness rates were high, reaching 100% in 13 specialties/areas of expertise: head and neck surgery, trauma surgery, general surgery–advanced program, hansenology, tropical medicine, emergency medicine, occupational medicine, radiotherapy, angiography and endovascular surgery, laparoscopic surgery, echocardiography, digestive endoscopy, and pediatric nephrology (Table 3).

Table 4 summarizes the Kolmogorov–Smirnov normality test and descriptive analysis of the indicators used as the dependent and independent variables.

Bivariate regression showed an association between the number of MRPs and the following variables: MHDI, GDP per capita, vulnerability index (GeoSES), number of general hospitals, number of specialized hospitals, number of teaching hospitals, number of primary healthcare units, number of undergraduate courses in medicine, rate of physicians per 100,000 inhabitants, and overall mortality rate per 100,000 inhabitants. The overall hospitalization rate per 10,000 inhabitants did not show a significant association in this analysis (Table 5).

The analysis of multicollinearity between model variables showed that the MHDI was excluded from the analysis due to autocorrelation with GeoSES (r = 0.904). GeoSES shows high vulnerability with the MHDI, but it has other dimensions, so it was chosen for analysis [16]. The total number of undergraduate courses showed multicollinearity with the total number of teaching units (r = 0.657), primary health care units (r = 0.735), general hospitals (r = 0.788), and specialized hospitals (r =0.804). Teaching units showed multicollinearity with the number of primary healthcare units (r = 0.742), general hospitals (r = 0.639), and teaching hospitals (r = 0.808). The number of primary healthcare units presented multicollinearity with the number of general hospitals (r = 0.808) and the number of specialized hospitals (r = 0.810). Finally, a strong correlation was found between the number of general and teaching hospitals (r = 0.769). Thus, five models with separate variables were adjusted, as shown in Table 6.

The models showed that with each increase of one unit of the vulnerability index (GeoSES), the number of MRPs increased, ranging from 8.122 (Model 5) to 11.138 (Model 4). With each increase in undergraduate degrees in medicine, the number of MRPs increased by 0.945 (Model 1). The number of physicians per 1000 inhabitants influenced the number of MRPs in Models (Models 3 and 4). With each increase of one physician per 1000 population, the number of MRPs increased from 0.537 (Model 3) to 0.845 (Model 5). With each increase in one unit of general hospitals, specialized hospitals, and teaching hospitals, the number of MRPs increased by 0.176 (Model 2), 0.168 (Model 3), and 0.022 (Model 5), respectively. With each increase in one unit of primary healthcare units, the number of MRPs increased by 0.032 (Model 5). Finally, with each increase of 1 death/100,000 inhabitants, the overall mortality rate increased, ranging from 0.006 (Model 5) to 0.022 (Model 4). The models recorded explanatory powers ranging from 81.7% (Model 4) to 90.3% (Model 5), based on R^2^ (Table 6).

## 4. Discussion

This study analyzed the panorama and contextual determinants (socioeconomic, structural, and epidemiological) of the number of MRPs in the northern region of Brazil. The results showed that only 3.6% of the cities had authorized MRPs. Most vacancies were concentrated in the state’s capital. Family and community medicine, pediatrics, medical clinics, gynecology, and obstetrics accounted for the largest number of MRPs and vacancies. The idleness rate in the investigated region was high. The contextual determinants of the number of MRPs were the vulnerability index (GeoSES), number of undergraduate courses in medicine, number of physicians per 1000 inhabitants, number of general hospitals, number of teaching units, number of primary healthcare units, and overall mortality rate.

This study showed that the vacancies were concentrated in basic specialties, mainly in family and community medicine. This profile is consistent with previous studies conducted in North Brazil and other regions [25,26]. Studies have shown that several policies and strategies implemented in Brazil have contributed to the creation or expansion of MRP vacancies in family and community medicine to help universalize vacancies in medical residency and strengthen primary healthcare (PHC) services [27]. These policies and strategies prioritized MRPs in family and community medicine due to the lack of specialists in this field all over the country [28]. The Primary Care Professional Valuation Program (in Portuguese Programa de Valorização do Profissional de Atenção Básica—PROVAB), PMM, and Pró-Residências stood out as key programs and policies to help expand MRPs. Furthermore, studies have shown that expanding the coverage and improving the infrastructure of PHC services in the country, as well as qualified and improved preceptorship integration into undergraduate courses and MRPs from other specialties, have contributed to expanding family and community medicine programs in Brazil [27].

The results showed that MRPs had a high idle rate, which was higher than that found for Brazil (29.3%), but similar to that observed for the North Region in 2019 (40.2%) [1]; this finding suggests MRPs idleness rate stability in this region. However, idleness rates were not homogeneous among North States; Pará State recorded the lowest idleness rate, whereas Rondônia, Acre, and Amapá had the highest idleness rates, which is consistent with a previous study conducted with data from 2019 [1]. Factors such as personal and contextual determinants are associated with MRPs idleness and physicians [29]. Factors associated with the academic infrastructure of MRP-offering institutions and municipalities [29] stood out, which may explain the differences in idleness rates observed between states. Studies have shown that proper workplace, better practice and work fields, sufficient physical resources and preceptors, and patients available for practical activities, among others, are factors determining MRPs idleness and professionals’ choice of these programs [29,30]. Pará State accounts for the largest number of medical schools, teaching units, and healthcare establishments in comparison to other states in the North Region [20]; this finding may explain its lower idleness rate than that of other states.

Family and community medicine had the highest idleness rates. This result has been reported in previous studies [1,31]. However, these studies did not analyze the idleness rate data in the North region based on medical specialties. Other studies have shown that some specialties are more attractive than others for residents, who are influenced by multiple factors, such as stability and quality of life; future income in the specialty; prospects for future work in the specialty, such as job opportunities and security; future practice location; personal determinants, such as sex and socioeconomic status; and educational factors such as undergraduate medical school type (public or private), previous educational and clinical experiences, and the infrastructure of the aforementioned locations [30]. These factors should be assessed in future studies to better understand the differences in idleness rates among different specialties and areas of expertise.

The multiple regression analysis conducted in the current study showed important contextual factors that determine the number of MRPs. Studies that focused on analyzing contextual factors associated with MRP offers in Brazil were not found in the literature. The data showed that the number of authorized MRPs increased with an increase in the vulnerability measured based on the socioeconomic index in GeoSES and in the number of physicians per 1000 inhabitants, the number of general specialized teaching units, and the number of primary health care units. These data indicate that cities with a higher availability of practice fields and higher socioeconomic status can influence MRP offers. More specifically, socioeconomic level is a proxy for higher income and provision of services in each region [16], and it can lead to increased availability of practice fields such as hospitals for MRPs implementation purposes.

There was an association between the rate of physicians per 1000 inhabitants and the number of MRPs, suggesting that the number of trained specialists working in the municipalities’ workforce increased [25] as the number of authorized MRPs increased. It was likely that the rate of physicians worked as a proxy for a human resources indicator associated with preceptorship and mentoring, which, in turn, were strongly associated with the number of MRPs [27]. There was a positive association between the number of medical schools and authorized MRPs. This finding suggests that the increase in medical schools in municipalities acts as an MRP opening booster and indicates the number of physicians who can act as preceptors and tutors in addition to practice fields, such as teaching hospitals, that are capable of favoring the opening and maintenance of these programs.

Finally, there was a positive association between the overall mortality rate and number of MRPs, suggesting that these MRPs have been offered in municipalities with higher mortality rates. Studies investigating the association between the overall mortality rate and number of MRPs were not found in the literature. However, cities recording the highest overall mortality rates in the North Region were represented by state capitals with the greatest infrastructure to offer MRPs, such as the number of hospitals and patient migration, which could explain this association. However, the opening and offering of MRPs may have occurred based on municipalities’ epidemiological needs [28], and this may explain the larger number of MRPs observed in cities with higher mortality rates.

This study had some limitations. The calculated MRPs indicators may be overestimated owing to flaws in programs’ status registration in the database. The adopted idleness rate indicator acted as a proxy for the true indicator because it did not include data on vacancies offered in 2022. Although some MRPs had authorized vacancies, they might not have been offered in the current year’s admission notice. We also did not perform a sensitivity analysis of the factors by specialty type. Specialties may have different contextual determinants. Finally, some variables could not be analyzed due to a lack of public data in the database, such as the MRPs financing type. The generalizability of the results to other geographic areas was limited because data from a single region in the country were analyzed.

This study has several strengths and contributions. To the best of our knowledge, the current study is the first to analyze contextual factors determining the number of authorized MRPs in the northern region; thus, it can be used to substantiate the analysis of factors capable of influencing the expansion and/or reduction in the number of MRPs in this region. In addition, the current study has analyzed the strengths and weaknesses in the distribution of MRP indicators based on state and specialty/area of expertise as well as the most and least attractive specialties/areas of expertise (the highest and lowest dropout rates, respectively) and those with the largest and smallest workforce in the assessed cities (the highest and lowest density per 100,000 inhabitants, respectively).

These data can substantiate both the expansion and direction to be taken in the opening of vacancies in the most needed specialties and areas of expertise. In addition, they can contribute to the formulation of both existing and new public policies focused on strengthening MRPs in the northern region. It is worth emphasizing that the current study can contribute to actions for the effective healthcare workforce dimensioning process to guarantee specialists’ training in priority municipalities, to develop residencies in networks of institutions, to improve residents’ training through Professional Master’s degree courses associated with residency, to organize integrated selection processes for all Brazilian regions, qualify care practice scenarios, acknowledge preceptorship activity, decentralize vacancy regulation and supervision to state medical residency commission, and to establish management information systems to help monitor MRPs indicators [1]. Increasing the number of MRPs—and, consequently, increasing human resources—in the northern region is feasible due to its number of medium- and large-sized hospitals; however, this must happen along with improvements in urbanization, security, technology, qualified preceptorship, and local infrastructure, among other contextual determinants analyzed in the current study.

## 5. Conclusions

This study showed that a small number of cities in the northern region have MRPs located mainly in the capital. Family and community medicine, pediatrics, internal medicine, gynecology, and obstetrics focused on the number of vacancies offered. Idleness rates were high, especially in the specialties of family and community medicine. The models showed an increase in the number of programs with an increase in the vulnerability index (GeoSES), rate of physicians per 1000 inhabitants, number of general hospitals, number of specialized hospitals, number of teaching units, number of primary healthcare units, and overall mortality rate.

## Figures and Tables

**Table 1:** Contextual (socioeconomic, structural, and epidemiological) indicators and data sources.

Indicators	Domain	Description	Data Source	Period
MHDI	Socioeconomic	It indicates the human development level of cities. It comprises three dimensions: longevity, education, and income. It ranges from 0 to 1; the closer the value is to 1, the higher the human development level.	IBGE Demographic Census [15]	2010
GDP per capita	Socioeconomic	Sum of production related to goods and services in the city divided by the number of inhabitants.	IBGE [15]	2017
GeoSES	Socioeconomic	It indicates the socioeconomic vulnerability of a given region. It has seven socioeconomic dimensions: education, mobility, poverty, wealth, income, segregation, and deprivation of resources and services. It ranges from −1 to +1; −1 is the worst socioeconomic context, whereas +1 is the best socioeconomic context.	GeoSES [16]	2020
Number of general hospitals	Structural	Absolute number of general hospitals in the city.	CNES [17]	2020
Number of specialized hospitals	Structural	Absolute number of specialized hospitals in the city.	CNES [17]	2020
Number of teaching hospitals	Structural	Absolute number of hospitals with teaching qualifications in the city.	CNES [17]	2020
Number of primary health care units	Structural	Absolute number of basic healthcare units in the city.	CNES [17]	2020
Number of undergraduate courses in medicine	Structural	Number of undergraduate courses in medicine in the city.	Inep [18]	2020
Rate of physicians per 1000 inhabitants	Structural	Number of physicians, divided by the number of inhabitants, multiplied by 100,000.	CNES [17]	2020
Overall mortality rate 100,000 inhabitants	Epidemiological	Number of deaths from all ICD-10 causes, divided by the number of inhabitants, multiplied by 100,000.	SIM [19]	2018
Overall hospitalization rate per 10,000 inhabitants	Epidemiological	Number of hospitalizations for all ICD-10 causes, divided by the number of inhabitants, multiplied by 10,000.	SIH-SUS [20]	2018

CNES: National Register of Health Establishments (in Portuguese, Cadastro Nacional dos Estabelecimentos de Saúde); IBGE: Brazilian Institute of Geography and Statistics (in Portuguese, Instituto Brasileiro de Geografia e Estatística); ICD-10: International Classification of DiseasesExternal 10th Revision; Inep: National Institute of Educational Studies and Research (in Portuguese, Instituto Nacional de Estudos e Pesquisas Educacionais Anísio Teixeira); GDP: Gross Domestic Product; GeoSES: Socioeconomic Index in the Geographic Context for Health Studies; MHDI: Municipal Human Development Index (in Portuguese, Índice de Desenvolvimento Humano Municipal); SIH-SUS: Hospitalization System of the Unified Health System (in Portuguese, Sistema de Informações Hospitalares do Sistema Único de Saúde); SIM: Mortality Information System (in Portuguese, Sistema de Informação sobre Mortalidade).

**Table 2:** Descriptive analysis of MRPs variables and indicators. North region, Brazil, 2022.

Variables/indicators	Total	State						
	North	Acre	Amazonas	Amapá	Pará	Rondônia	Roraima	Tocantins
Number of MRPs *	295 (100.0%)	18 (6.1%)	75 (25.4%)	10 (3.4%)	100 (33.9%)	36 (12.2%)	11 (3.7%)	45 (15.3%)
Number of institutions with MRPs *	55 (100.0%)	3 (5.5%)	18 (32.7%)	4 (7.3%)	10 (18.2%)	11 (20.0%)	1 (1.8%)	8 (14.5%)
Total number of vacancies in MRPs *	2.261 (100.0%)	182 (8.0%)	729 (32.2%)	101 (4.5%)	911 (40.3%)	325 (14.4%)	107 (4.7%)	306 (13.5%)
Number of vacancies of R1 *	983 (100.0%)	67 (6.8)	271 (27.6)	33 (3.4)	334 (34.0)	122 (12.4)	39 (4.0)	117 (11.9)
Number of vacancies occupied/registered residents *	1.622 (100.0%)	106 (6.5%)	408 (25.2%)	49 (3.0%)	672 (41.4%)	175 (10.8%)	49 (3.0%)	163 (10.0%)
Unoccupied vacancies *	1.039 (100.0%)	76 (7.3%)	321 (30.9%)	52 (5.0%)	239 (23.0%)	150 (14.4%)	58 (5.6%)	143 (13.8%)
Idle rate (%)	46.0	41.8	44.0	51.5	26.2	46.2	54.2	46.7
Density of authorized vacancies per 100,000 inhabitants	11.9	20.4	16.8	11.6	10.4	17.2	19.0	18.8
Density of authorized vacancies of R1 per 100,000 inhabitants	5.2	7.5	6.2	3.8	3.8	6.5	6.9	7.2
Density of residents per 100,000 inhabitants	8.5	11.9	9.4	5.6	7.6	9.3	8.7	10.0

R1: Number of authorized places for the first year of residence; * absolute number and percentage in parentheses in relation to the total distribution of states in the northern region.

**Table 3:** Overview of indicators of medical residency programs based on specialty or areas of expertise. North region, Brazil, 2022.

Specialties/Areas of Expertise	Authorized Vacancies, *n* (%)	R1, *n* (%)	Enrolled Residents, *n* (%)	Idle Vacancies	Density of Vacancies *	Density of Residents *	Idleness Rate (%)
Specialties							
Anesthesiology	153 (6.77)	51 (5.19)	128 (7.89)	25	0.81	0.27	16.34
Cardiology	30 (1.33)	15 (1.53)	25 (1.54)	5	0.16	0.08	16.67
Cardiovascular surgery	23 (1.02)	5 (0.51)	9 (0.55)	14	0.12	0.03	60.87
Hand surgery	4 (0.18)	2 (0.20)	3 (0.18)	1	0.02	0.01	25.00
Head and neck surgery	6 (0.27)	3 (0.31)	0 (0.00)	6	0.03	0.02	100.00
Digestive system surgery	18 (0.80)	9 (0.92)	8 (0.49)	10	0.09	0.05	55.56
Trauma surgery	12 (0.53)	0 (0.00)	0 (0.00)	12	0.06	0.00	100.00
General surgery	192 (8.49)	64 (6.51)	124 (7.64)	68	1.01	0.34	35.42
General surgery—advanced program	4 (0.18)	2 (0.20)	0 (0.00)	4	0.02	0.01	100.00
Pediatric surgery	6 (0.27)	2 (0.20)	6 (0.37)	0	0.03	0.01	0.00
Plastic surgery	6(0.27)	2 (0.20)	2 (0.12)	4	0.03	0.01	66.67
Thoracic surgery	4 (0.18)	2 (0.20)	1 (0.06)	3	0.02	0.01	75.00
Vascular surgery	10 (0.44)	5 (0.51)	6 (0.37)	4	0.05	0.03	40.00
Medical clinic	292 (12.91)	146 (14.85)	237 (14.61)	55	1.54	0.77	18.84
Dermatology	48 (2.12)	16 (1.63)	46 (2.84)	2	0.25	0.08	4.17
Endocrinology and metabolism	14 (0.62)	7 (0.71)	12 (0.74)	2	0.07	0.04	14.29
Endoscopy	8 (0.35)	4 (0.41)	4 (0.25)	4	0.04	0.02	50.00
Gastroenterology	10 (0.44)	5 (0.51)	4 (0.25)	6	0.05	0.03	60.00
Geriatrics	12 (0.53)	6 (0.61)	4 (0.25)	8	0.06	0.03	66.67
Gynecology and Obstetrics	276 (12.21)	92 (9.36)	202(12.45)	74	1.45	0.48	26.81
Hansenology	1 (0.04)	0 (0.00)	0 (0.00)	1	0.01	0.00	100.00
Hematology and hemotherapy	1 (0.53)	6 (0.61)	5 (0.31)	7	0.06	0.03	58.33
Hepatology	8 (0.35)	0 (0.00)	2 (0.12)	6	0.04	0.00	75.00
Infectiology	75 (3.32)	25 (2.54)	43 (2.65)	32	0.40	0.13	42.67
Mastology	10 (0.44)	5 (0.51)	3 (0.18)	7	0.05	0.03	70.00
Family and community medicine	404 (17.87)	202 (20.55)	172 (10.60)	232	2.13	1.06	57.43
Intensive care medicine	102 (4.51)	34 (3.46)	35 (2.16)	67	0.54	0.18	65.69
Tropical medicine	2 (0.09)	0 (0.00)	0 (0.00)	2	0.01	0.00	100.00
Nephrology	28 (1.24)	14 (1.42)	13 (0.80)	15	0.15	0.07	53.57
Neurosurgery	37 (1.64)	7 (0.71)	23 (1.42)	14	0.19	0.04	37.84
Neurology	12 (0.53)	4 (0.41)	12 (0.74)	0	0.06	0.02	0.00
Ophthalmology	90 (3.98)	30 (3.05)	47 (2.90)	43	0.47	0.16	47.78
Surgical oncology	33 (1.46)	11 (1.12)	11 (0.68)	22	0.17	0.06	66.67
Clinical oncology	24 (1.06)	9 (0.92)	9 (0.55)	15	0.13	0.05	62.50
Orthopedics and traumatology	105 (4.64)	35 (3.56)	66 (4.07)	39	0.55	0.18	37.14
Otorhinolaryngology	21 (0.93)	7 (0.71)	22 (1.36)	−1 **	0.11	0.04	−4.76 **
Pathology	18 (0.80)	6 (0.61)	7 (0.43)	11	0.09	0.03	61.11
Pediatrics	275 (12.16)	92 (9.36)	207 (12.76)	68	1.45	0.48	24.73
Pulmonology	4 (0.18)	2 (0.20)	4 (0.25)	0	0.02	0.01	0.00
Psychiatry	57 (2.52)	19 (1.93)	26 (1.60)	31	0.30	0.10	54.39
Radiology and diagnostic imaging	51 (2.26)	17 (1.73)	39 (2.40)	12	0.27	0.09	23.53
Rheumatology	12 (0.53)	6 (0.61)	10 (0.62)	2	0.06	0.03	16.67
Urology	24 (1.06)	8 (0.81)	18 (1.11)	6	0.13	0.04	25.00
Emergency medicine	9 (0.40)	3 (0.31)	0 (0.00)	9	0.05	0.02	100.00
Occupational medicine	4 (0.18)	2 (0.20)	0 (0.00)	4	0.02	0.01	100.00
Radiotherapy	4 (0.18)	1 (0.10)	0 (0.00)	4	0.02	0.01	100.00
Areas of expertise							
Angiography and endovascular surgery	2 (0.09)	0 (0.00)	0 (0.00)	2	0.01	0.00	100.00
Laparoscopic surgery	5 (0.22)	0 (0.00)	0 (0.00)	5	0.03	0.00	100.00
Echocardiography	2 (0.09)	0 (0.00)	0 (0.00)	2	0.01	0.00	100.00
Digestive endoscopy	1 (0.04)	0 (0.00)	0 (0.00)	1	0.01	0.00	100.00
Pediatric hematology and hemotherapy	4 (0.18)	0 (0.00)	1 (0.06)	3	0.02	0.00	75.00
Hemodynamics and interventional cardiology	4 (0.18)	0 (0.00)	1 (0.06)	3	0.02	0.00	75.00
Pediatric intensive care medicine	18 (0.80)	0 (0.00)	7 (0.43)	11	0.09	0.00	61.11
Pediatric nephrology	4 (0.18)	0 (0.00)	0 (0.00)	4	0.02	0.00	100.00
Neonatology	59 (2.61)	0 (0.00)	11 (0.68)	48	0.31	0.00	81.36
Pediatric cardiology	4 (0.18)	0 (0.00)	3 (0.18)	1	0.02	0.00	25.00
Pediatric intensive care	18 (0.80)	0 (0.00)	7 (0.43)	11	0.09	0.00	61.11
Pediatric neurology	4 (0.18)	0 (0.00)	4 (0.25)	0	0.02	0.00	0.00
Neurorradiology	4 (0.18)	0 (0.00)	1 (0.06)	3	0.02	0.00	75.00

R1: Number of authorized places for the first year of residence; * per 100,000 inhabitants; ** negative numbers due to a resident enrolled greater than the number of vacancies in the specialty.

**Table 4:** Descriptive analysis of number of MPRs and contextual (socioeconomic, structural, and epidemiological).

Indicators	K-S Test (*p* Value)	Mean	Standard Deviation	Median	P25–P75	Min–Max
Dependent variable						
Number of MRPs	0.510 (<0.001)	0.66	5.67	0	0; 0	0; 80
Independent variables						
MHDI	0.035 (0.200)	0.60	0.06	0.60	0.57; 0.65	0.42; 0.79
GDP per capita (R$)	0.141 (<0.001)	14.75	8.69	12.58	9.30; 17.23	4.60; 70.52
GeoSES	0.035 (0.200)	−0.64	0.17	−0.64	−0.76; −0,54	−1.00; 0,05
Number of general hospitals	0.368 (<0.001)	1.08	2.07	1	0; 1	0; 22
Number of specialized hospitals	0.498 (<0.001)	0.15	1.23	0	0; 0	0; 19
Number of teaching hospitals	0.453 (<0.001)	1.17	9.95	0	0; 0	0;185
Number of primary health care units	0.281 (<0.001)	6.66	11.50	4	2; 7.3	0; 158
Number of undergraduate courses in medicine	0.530 (<0.001)	0.07	0.40	0	0; 0	0; 4
Rate of physicians per 1000 inhabitants	0.189 (<0.001)	0.56	0.51	0.29	0.20; 0.40	0; 4.07
Overall mortality rate 100,000 inhabitants	0.039 (0.098)	482.64	130.14	396.68	318.29; 476.02	138.54; 981.14
Overall hospitalization 10,000 inhabitants	0.051 (0.007)	526.75	249.18	348.84	226.51; 505.39	32.63; 1822.98

Note: All statistics used for the unit of analysis for cities in the northern region (n = 450), except MHDI for one, due to missing data. GDP: Gross Domestic Product; K-S: Kolmogorov–Smirnov test; MHDI: Max: Maximum; Min: Minimum; Municipal Human Development Index (in Portuguese, Índice de Desenvolvimento Humano Municipal); P25: 25th percentile; P75: 75th percentile.

**Table 5:** Bivariate analysis of the relationship between contextual factors (socioeconomic, structural, and epidemiological) and the number of MRPs. Factors associated with the number of MRPs. North region, Brazil, 2022.

Indicators	β	95%CI	*p* Value
MHDI			
GDP per capita (R$)	5.323	1.996; 8.651	0.002
GeoSES	9.989	7.960; 11.819	<0.001
Number of general hospitals	0.319	0.277; 0.362	<0.001
Number of specialized hospitals	0.320	0.237; 0.368	<0.001
Number of teaching hospitals	0.030	0.026; 0.035	<0.001
Number of primary health care units	0.041	0.034; 0.048	<0.001
Number of undergraduate courses in medicine	1.586	1.413; 1.760	<0.001
Rate of physicians per 1000 inhabitants	1.370	1.032; 1.708	<0.001
Overall mortality rate per 100,000 inhabitants	0.005	0.003; 0.007	<0.001
Overall hospitalization rate per 10,000 inhabitants	0.000	−0.001; 0.001	0.840

GDP: Gross Domestic Product; GeoSES: Socioeconomic Index in the Geographic Context for Health Studies; MHDI: Municipal Human Development Index (in Portuguese, Índice de Desenvolvimento Humano Municipal); β = regression coefficient; 95%CI = 95% Confidence Interval.

**Table 6:** Factors associated with the number of MRPs offered. North region, Brazil, 2022.

Indicators	β	95%CI	*p* Value
Model 1			
GDP per capita (R$)	−0.017	−0.055; 0.021	0.380
GeoSES	8.173	4.750; 11.596	<0.001
Number of undergraduate courses in medicine	0.945	0.734; 1.155	<0.001
Rate of physicians per 100,000 Inhabitants	−0.057	−0.539; 0.425	0.816
Overall mortality rate	0.008	0.003; 0.012	0.002
R^2^: 0.884			
Model 2			
GDP per capita (R$)	0.008	−0.025; 0.040	0.648
GeoSES	8.173	5.043; 11.302	<0.001
Number of general hospitals	0.176	0.135; 0.217	<0.001
Rate of physicians per 100,000 inhabitants	0.063	−0.274; 0.413	0.725
Overall mortality rate	0.010	0.006; 0.015	<0.001
R^2^: 0.874			
Model 3			
GDP per capita (R$)	−0.011	−0.028; 0.006	0.194
GeoSES	8.884	7.575; 10.193	<0.001
Number of specialized hospitals	0.168	0.145; 0.178	<0.001
Rate of physicians per 100,000 inhabitants	0.537	0.3139; 0.736	<0.001
Overall mortality rate	0.007	0.005; 0.009	<0.001
R^2^: 0.866			
Model 4			
GDP per capita (R$)	−0.015	−0.051; 0.017	0.362
GeoSES	11.138	9.896; 12.379	<0.001
Number of teaching hospitals	0.022	0.019; 0.025	<0.001
Rate of physicians per 100,000 inhabitants	0.736	0;566; 0.906	0.725
Overall mortality rate	0.012	0.010; 0.015	<0.001
R^2^: 0.817			
Model 5			
GDP per capita (R$)	−0.018	−0.054; 0.020	0.379
GeoSES	8.122	6.135; 11.888	<0.001
Number of primary health care units	0.032	0.255; 0.039	<0.001
Rate of physicians per 100,000 inhabitants	0.845	0.479; 1.189	<0.001
Overall mortality rate	0.006	0.003; 0.009	<0.001
R^2^: 0.903			

Note: Number of model observations (n = 450 [number of municipalities in northern states]). GDP: Gross Domestic Product; GeoSES: Socioeconomic Index in the Geographic Context for Health Studies; MHDI: Municipal Human Development Index (in Portuguese, Índice de Desenvolvimento Humano Municipal); β = regression coefficient; 95%CI = 95% Confidence Interval; R^2^ = Coefficient of determination.

## Data Availability

Not applicable.
